# Pilot implementation of a co-mentoring circles program for the clinical research professionals: Evidence for formative evaluation and logic model

**DOI:** 10.1017/cts.2023.712

**Published:** 2024-01-05

**Authors:** July D. Nelson, Mendy E. Dunn, Yulia A. Levites Strekalova

**Affiliations:** 1 Department of Health Services Research, Management and Policy, College of Public Health and Health Professions, University of Florida, Gainesville, FL, USA; 2 UF-FSU Clinical and Translational Science Institute, University of Florida, Gainesville, FL, USA

**Keywords:** Clinical research professionals, translational research, mentoring, workforce development

## Abstract

Clinical research professionals (CRPs) are essential contributors to clinical and translational research endeavors, encompassing roles such as research nurses, research coordinators, data managers, and regulatory affairs specialists. This paper reports on the implementation of a novel training program for the CRPs, the Co-mentoring Circles Program, developed by the University of Florida Health Clinical Research Professionals Consortium, and proposes an initial logic model of CRP workforce development informed by the observations, participant feedback, and the established Translational Workforce Logic Model. The co-mentoring program was delivered through an online didactic curriculum and bi-monthly meetings over nine months, from January to September 2022. The formative evaluation identified the factors that support CRP workforce development through knowledge acquisition and professional relationship building. Finally, this paper proposes a logic model of CRP workforce development, including financial and human inputs, didactic and co-mentoring activities, workforce outputs, outputs related to workforce and clinical research study progress, and resulting impacts of increased national capacity for translational research and increased rate of research translation.

## Introduction

Clinical research professionals (CRPs) are essential contributors to clinical and translational research endeavors, encompassing roles such as research nurses, research coordinators, data managers, and regulatory affairs specialists. These non-faculty members assume pivotal responsibilities within research studies, playing a critical part in the regulatory and research dimensions that significantly shape study quality, operational efficiency, and the safety of participants. The increasing number and complexity of clinical trial protocols have generated an unrelenting demand for proficient CRPs, a demand that consistently surpasses available resources. This demand underscores the urgent need to cultivate and sustain a skilled and seasoned CRP workforce that supports the expansive clinical research domain [1]. Meanwhile, high staff turnover at academic medical centers endangers the sustainability of the CRP workforce. New CRPs need mentoring to build their skills and understanding of complex organizational environments. Experienced CRPs who could support the development of more junior colleagues are overtaxed, and their roles do not formally incentivize or protect time for mentoring. Furthermore, established CRPs represent an important but underutilized reservoir of institutional knowledge within academic medical centers. These CRPs prove instrumental in facilitating the assimilation of recruits and infusing clinical trials with insights that inform study design and execution. The total wealth of this brain trust can only be realized when new and experienced CRPs are provided with a supportive space to exchange knowledge and build lasting relationships. While CTSAs have invested substantially in developing workforce development programs for trainees and faculty, the conceptual and applied efforts around workforce development for CRPs remain scarce. Therefore, there are critical gaps in both practical and conceptual efforts in support of the professional workforce development for CRPs. To contribute to both of these areas, this paper reports on the implementation of a novel training program for the CRPs, the Co-mentoring Circles Program, developed by the University of Florida Health Clinical Research Professionals Consortium (UF CRPC), and proposes an initial logic model of CRP workforce development informed by the observations, participant feedback, and the established Translational Workforce Logic Model [2].

### Mentoring theory for CRP workforce development

Successful mentoring is rooted in a relationship between the mentor and the mentee. Conceptually, the relational-cultural theory (RCT) provides a valuable framework for understanding mentoring relationships. The RCT proposes that people grow through and towards relationships and that development is a mutual exchange through which all involved parties contribute, grow, and benefit [3]. Traditionally, in a mentoring relationship, there is a clear boundary between the mentor and the mentee, where mentors are expected to provide support, encouragement, and guidance while mentees familiarize themselves with new tasks [4]. However, by shifting the focus on development through growth-fostering relationships, mentors and mentees can be afforded development opportunities through co-mentoring. At its core, co-mentoring or collaborative mentoring is a supportive assistance provided by several connected individuals who can act as teachers, counselors, and demonstrators for each other [5]. Active engagement in reciprocal learning reconfigures power dynamics to endorse a principle of equality [6]. This dynamic configuration renders the relationships among participants nonhierarchical, mutual, and reciprocally nurturing. While co-mentoring unites individuals in a mutually beneficial relationship, it also focuses on career and psychological development. Most importantly, there is evidence that a culture or expectation of mentoring is often lacking in clinical and translational science [7,8].

### Program theory for CRP workforce development

Program theory and logic models are essential tools for strategic planning, evaluation, and continuous development of workforce training initiatives. Within the translational workforce context, Rubio and colleagues proposed a logic model to design and assess the success of translational training programs [2]. While basic research and clinical research domains are underpinned by established definitions, translational research, until recently, lacked a standardized characterization. This implied that specifying critical components such as a suitable curriculum, specific program objectives, and clear competencies expected from trainees has been a daunting task for a long time. A working definition of translational research became necessary to establish the translational workforce logic model. Accordingly, for Rubio and colleagues, translational research fosters the multidirectional integration of basic research (T1), patient-oriented research (T2), and population-based research (T3), with the long-term aim of improving the health of the public [2]. As with most logic models, the translational workforce logic model emphasizes inputs (financial and human resources), activities (didactic coursework, mentored research, research collaborations, and practicums), outputs (trained researchers), and outcomes: short-term (training changes achieved through trainee and faculty satisfaction, innovative and ethically designed studies, and competitive grant proposals), intermediate-term (practice changes achieved through study implementation, effective human subject use, collaborative research, and published manuscripts), and long-term (impact realized through the increased capacity for translational research and improved human health status). The model provides a template for conducting rigorous evaluation to document how the program meets its objectives. However, it is focused on the experiences and outcomes of the PhD trainees. It lacks the context specific to the CRP workforce development resources, activities, and outcomes in achieving a shared impact of increased research capacity and contributions to improved human health.

## Practical implementation of a co-mentoring program for CRP workforce development

### Description

The UF CRPC, supported by the Clinical and Translational Science Institute, implemented the co-mentoring training pilot program in 2022. The program’s overarching goal was to provide CRPs with foundational training specifically focused on operational and regulatory elements necessary for the ethical conduct of clinical trials. The program also pursued the specific objectives of (1) provide learners with a foundation that addresses and expands beyond the core knowledge about human subjects research (HSR) protections and Good Clinical Practice (GCP); (2) certify institutional research professionals to a baseline core knowledge foundation; (3) prepare participants for national professional certification exams; (4) create supportive mentoring opportunities facilitating the integration of core concepts relevant to specific needs of CRPs across the academic health center; and (5) prepare the next generation of peer mentors. The program consisted of asynchronous online coursework and monthly small group activities, which participants attended physically or virtually. At completion, participants received a certificate and also logged in their institutional personal training record.

### Participant recruitment

Participants were eligible to participate in a co-mentoring circle if they had been employed at the UF for a minimum of 6 months, had completed all mandated institutional training (e.g., HIPAA, IRB) for Human Subjects Research, were committed to developing skills for career development, and were working with human subjects in biomedical, social/behavioral, or education research at the time of recruitment. Participants also needed to confirm that they were intentional about getting their certification and that they would be taking the certification exam in the near future. Invitations to apply for the training were sent to the institution-wide Clinical Research Professionals Consortium (CRPC) listserv on October 4, 2021. Prospective applicants were encouraged to apply to be a part of a future model for professional engagement with high professional standards. Participants submitted a statement of interest in the program (max. 500 words), their resume, and a letter of support from their supervisor as supporting documents. Information about the department, job title, degree(s), field of study, and primary CRP responsibilities were also captured. Preference was given to applicants with Clinical Research Coordinator or Research Nurse job descriptions. Facilitators were CRPs known for the leadership in their current professional roles or through the CRP national organization or chapter leadership. In total, forty-nine participants were recruited. Five participants had dropped out over the 9-month implementation period, two of which had scheduling conflicts with clinical schedules and study activities. Three participants left the institution.

### Planning and implementation

To address its first three objectives of providing CRPs with the core human subjects protection and GCP knowledge and preparing them for national certification, the co-mentoring program utilized the ACRP professional development e-learning platform, which offers a specific curriculum for training clinical and research staff. See Appendix A for a listing of the didactic modules. The online platform provided a performance-based, interactive learning system to support knowledge transfer into performance. It incorporated knowledge checks and offered a customizable learning experience. Co-mentoring facilitators also used it to track and audit eLearning performance by the co-mentoring participants to identify knowledge gaps within the group and guide circle discussions.

To address the two mentoring objectives, the program organized co-mentoring circle meetings. With participants located at various clinics and offices across campus and given the nature of busy patient-facing clinical research schedules, virtual meeting technology was leveraged for co-mentoring circle sessions. Some groups offered optional periodic meet-and-greets at central locations and final wrap-up celebrations. Co-mentoring was delivered through bi-monthly meetings from January to September 2022. Participants were assigned across seven groups of co-mentoring circles, and group sizes ranged from five to ten members. Participants were expected to cover all of the didactic materials by the end of the training program. The estimated time commitment for the mentoring meetings was 4-5 hours per month.

## Program results and formative evaluation

To collect formative evaluation data and assess the contribution of the co-mentoring circles program to the development of the CRP workforce, program leaders engaged the UF CTSI evaluation team in conducting an online focus group approved as a quality improvement study. A facilitator external to the co-mentoring program but familiar with the clinical research process and institutional research infrastructure conducted the interview (Please see Appendix B: Interview Guide). Eleven participants attended the focus group. Several co-mentoring facilitators participated in the focus group. The conversation was recorded, transcribed, and content-analyzed using a-priori and emergent codes. Using a-priori codes is a valid technique for analyzing qualitative data [9]. The four a-priori codes originated from the Partnership Outcome Spaces Framework, which provides a structure to plan for preferred outcomes in transdisciplinary projects. The framework comprises four pillars: situation, knowledge, learning, and relationships [10,11]. These four pillars were used as a-priori codes. The situation is perceived as the challenge that the partnership seeks to address. Knowledge refers to generating relevant knowledge flows, including scholarly knowledge and other societal knowledge forms, and making them accessible and meaningful to researchers, participants, and beneficiaries. Learning pertains to mutual and transformational understanding by researchers and research participants to increase the likelihood of persistent change. Relationships emphasize the evolution of relationships through the process of the partnership [10,11]. These four a-priori codes were used as first-level codes and applied to organize participant feedback. Next, emergent second-level codes were developed to capture the details of participant elaborations on the co-mentoring circles experience. The second-level codes addressed the following questions that guided the formative evaluation: What situation needs to change? What can enhance knowledge? What learning value does the program bring? How does the program foster relationships? Table 1 below is a synthesis of the codebook.

**Table 1.** Codebook with a-priori and emergent codes used to analyze participant feedback

First-level codes	Second-level codes
**Situation** The challenge that participants seek to address and/or an improvement within the situation or field of inquiry	**What is the situation that needs to change?** Inclusion of PIs in sessions Participants distribution within groups Program conducted in fewer weeks Better documentation Co-mentoring for early coordinators Commitment of PI to invest in staff Materials for certification preparation New topics for future co-mentoring circles
**Knowledge** The generation of relevant stocks and flows of knowledge, including scholarly knowledge and other societal knowledge forms, and making those insights accessible and meaningful to others	**What can enhance knowledge?** Certification Mentoring SOPs Professional task rehearsal Working for problem-solving
**Learning** The mutual and transformational learning to increase the likelihood of persistent change	**What learning value does the program bring?** Application with topics Group discussions Safe space to learn Curriculum enhancement Transitioning between roles Reading materials
**Relationships** The evolution of relationships through the process of partnership	**How does the program foster relationships?** Meeting outside of the workplace Networking Professional identity

PIs: Principal Investigator; SOPs: Standard Operating Procedures.

The ***situation*** is that CRPs are vital contributors to translational research. Yet, workforce development efforts contributing to the sustainability of the CRP workforce are lacking. Results from this training program corroborated this point as participants pointed out that their Principal Investigators (PIs) neither supported nor encouraged them to participate in the program. Additionally, there was a consensus that program materials should be pertinent to the certification exam, especially for participants who still need certification. As a result, exposure to additional materials such as test questions or exposure to people’s experiences on taking the certification exam became a recurrent theme. One participant specifically mentioned that she would like to know people’s experiences of the testing environment, including whether the materials used in the training were relevant and comparable to what was on the exam.

Collectively, participants indicated that from a ***knowledge*** perspective, they felt more comfortable in their professional tasks after they had attended the program. Four specific topics encouraged such confidence. First, the ethics and human subject protection. As noted by a participant, this topic stood out because it was a general reminder of ethical principles to follow when working with patients. Another participant pointed out that the session on Ethics was “*also a good point for discussion and sharing experiences across the group.”* Second, introduction to clinical trials. Participants found it helpful to know more about this topic as it helped review the fundamentals of clinical trials. One participant highlighted that the “*Introduction to Clinical Trials had the most useful information untouched in other modules, but it was more comprehensive than any other.”* Third, risk-based monitoring. The idea of being presented with content they were not familiar with was also another factor that increased participants’ confidence. For instance, one participant shared that risk-based monitoring piqued her curiosity as she was unfamiliar with the topic. It made her contemplate the discipline more because it seemed like *“a natural progression from Clinical Research Coordinator.”* Fourth, critical skills for ensuring quality control and site quality management tools, including SOPs, metrics, and training, were also among topics that participants found helpful, mainly because of how applicable they are to their professional tasks.

What made the ***learning*** process mutual and transformational was the presentation mode of the training program. Some participants preferred the online sessions to the in-person sessions. Additionally, the majority agreed that the impact of the co-mentoring circles discussions was far more palpable than the didactic modules/curriculum they were taught. For instance, one participant commented that she was more confident in her professional abilities from the co-mentoring circles, although “*the curriculum was good*.” She also appreciated the other mentees sharing difficulties regarding donor procedures in their respective departments, as “*this can be intimidating*.” The opportunity to have a safe space for sharing ideas and gaining insights from people’s experiences was also a highlight of the program. A participant shared that as a new coordinator, she felt welcomed to be *“in a group that had different levels of experiences.”* This feeling was echoed by other participants who shared that they *“enjoyed the networking aspect in the group and seeing how other departments do things.”* More importantly, they appreciated the opportunity to “*bounce ideas and to serve as an outlet for people who had questions and who may not have felt comfortable going to their department.”*

Participants unanimously acknowledged that the program was critical in fostering ***relationship*** building, developing their networking ability beyond their respective departments, and supporting their professional identity. All highlighted this opportunity to learn from other professionals even after the program had ended because networking *“brought a sense of community.”* One participant who attended the program virtually from Jacksonville highlighted that it was “*nice to build a network with Gainesville and Jacksonville because it’s often very separate in so many aspects.”* Another participant remained adamant about this aspect of the program because networking is “*vital as you progress through your career when you decide what your next level should be or in.”*

### Program-based logic model of CRP workforce development

This co-mentoring circle training program sheds new light on the components necessary for the success of programs targeting CRPs and their professional development. Accordingly, we developed the Clinical Research Professional Logic Model based on the data collected through the Focus Group (Table 2).

**Table 2.** The logic model of clinical research professional workforce development

Inputs	Activities	Outputs	Short-term outcomes (Training changes)	Intermediate-term outcomes (Practice changes)	Long-term outcomes (Impact)
**Financial inputs:** Institutional investments; PIs funding for CRPs to engage in training. **Human resources:** new CRPs, experienced CRPs, institutional champions to promote CRP development and training content providers.	**Didactic coursework** focused on professional skills, recognized competencies, and certification preparation. In-person and virtual guided discussions Co-mentoring and network events that promote community and professional identity development	**Well-trained and well-mentored CRPs** who are effectively onboarded to their job responsibilities CRP national certification CRPs who become mentors to new CRPs	New and experienced CRP satisfaction Increased professional skills Effective initiation of studies	Successful career development measured by increased rate of CRP retention, engagement in professional societies, and organizational leadership Effective conduct of clinical and translational studies without deviations or violations	Increased national capacity for translational research and increased rate of research translation

CRPs: Clinical Research Professionals; PIs: Principal Investigators.

Inputs include the tools and resources (both human and financial) that are put in place to achieve specific pre-established objectives about CRPs. Activities encompass the tasks/learning activities in which CRPs engage, which can be conducted in person or virtually. Outputs pertain to the CRP’s ability to network outside their academic department and feel safe while discussing topics they were unfamiliar with. Short-term outcomes encompass the evidence of improvement over time in the CRP knowledge and skills, following the implementation of the gained knowledge into the initiation and implementation of clinical studies. Intermediate-term outcomes pertain to the evidence of successful career development, as measured by the ability to achieve leadership positions within and beyond the home institution. Finally, the long-term outcomes for the CRPs fully align with those for the other groups (i.e., translational trainees and early-career faculty) [2] and include the increased national capacity for translational research and increased rate of research translation.

## Discussion and lessons learned

The co-mentoring program for CRPs was implemented during the COVID-19 pandemic, which proved opportune for study coordinators due to the available time. The essence of RCT provides a valuable framework for this training program, accentuating the value of reciprocal exchange. The program used the ACRP curriculum which allowed the research professionals to access robust and standardized content. The ACRP curriculum provided an essential foundation. However, we also found that integration of the local context is critical to ensure the relevance of training to job tasks, promote continuous engagement with the overall program, co-create learning teams, and support relationship building among the research professionals. Furthermore, the logic model presented above provides a framework for planning and evaluating workforce development efforts for research professionals linking short-term training experience, on-the-job performance, and retention within the research coordination profession.

This program aimed to achieve specific outcomes, but it also had ripple effects beyond its original focus on supporting professional development at one academic medical center. Firstly, the program developed a specific curriculum for mentoring and certification preparation. It enhanced local approaches to developing and delivering professional development curricula to prepare study coordinators for national certification. The structured curriculum provided explicit knowledge on best practices in managing clinical research. At the same time, the peer conversations facilitated the sharing of tacit knowledge and understanding, often acquired over a more extended period. Over time, we hope this can lead to a conscious choice of research coordination as a profession. Additionally, we aspire for programs like this to raise awareness of the profession and recognize the skills, expertise, and capabilities accumulated by CRPs.

Secondly, mentoring as a topic was incorporated into other training programs targeted at research coordinators, students, research disciplines, postdocs, and junior faculty. For instance, the Responsible Conduct of Research program implemented at the University of Florida in 2021 included a mentoring component, which was well-received and attended by study coordinators. Moreover, co-mentoring programs began to emerge at the national level, and implementing co-mentoring circles became a method for creating and disseminating shared knowledge among clinical research coordinators. As evidenced in the narratives of focus group participants, the training initiative has catalyzed personal growth and fortified the bonds they have developed. This underlines the imperative of nurturing growth-enabling relationships, an essential facet for cultivating learning and advancement within the discrete realms of CRPs. Yet, the continuous efforts around CRP workforce development must be contextualized within a broader clinical and translation research ecosystem. Understanding the roles of CRPs within this system and discerning the factors that obstruct CRP workforce development serves as the compass guiding the formulation of forthcoming programs akin to the present endeavor.

Integral to this ecosystem of clinical and translational research is culture, an element we gleaned from the focus group discussions. An evident dearth of a culture of learning and mentoring within their units emerged, with participants recounting the absence of encouragement or backing from their PIs upon enrolling in the program. This raises concerns about the alignment between acquired insights and their practical application within CRPs’ respective units. A plausible remedy, suggested by participants, involves the involvement of PIs in forthcoming co-mentoring circle initiatives, paving the way for a synchronized growth journey among all stakeholders. While expecting PIs to attend all sessions might be unfeasible, their pivotal role in instigating change and progression renders their inclusion crucial for meaningful deliberations and impactful commitments.

This program also had cultural and relational implications. Study coordinators engaged in conversations with their principal investigators to gain support for attending the sessions. Although some of these conversations were challenging, the recognition of the need for professional development and support by CTSI leadership started to shift the institutional culture regarding recognizing CRPs as vital research workforce. Furthermore, the peer-to-peer interactions fostered enculturation and knowledge sharing among study coordinators. Most importantly, co-mentoring circles created opportunities for peer-to-peer interactions and establishing peer networks. These interactions and joint advocacy contributed to the recognition of clinical research as a professional career. The ripple effects of this recognition and professionalization offer younger professionals the chance to perceive clinical research as a deliberate career path rather than a happenstance.

Divergent cohorts of individuals constitute the CRP workforce, each stationed at distinct junctures in their professional trajectories. Some are more experienced and have acquired the necessary knowledge and skills about the institution’s processes and procedures. Others are less experienced and demand systematic onboarding to the institutional and professional expertise tailored to their job tasks. Therefore, the blueprint for future co-mentoring circle programs must align with the specific demands of the target audiences. While we are not currently collecting additional long-term data, we recognize the need to do so to enhance our logic model to better guide future workforce development efforts. For the next generation of CRPs, mentored training is anticipated to be critical to rapidly cultivate essential skills. Workforce development efforts for clinical research can be strengthened practically by implementing workforce development programs that focus on the unique needs of CRPs. They can also be strengthened conceptually by guiding the program development and evaluation of such workforce development efforts.

## Supporting information

Nelson et al. supplementary material 1 Nelson et al. supplementary material

Nelson et al. supplementary material 2 Nelson et al. supplementary material

## Supplementary material

The supplementary material for this article can be found at https://doi.org/10.1017/cts.2023.712.
