# Supplementary material for: Pilot implementation of a co-mentoring circles program for the clinical research professionals: Evidence for formative evaluation and logic model
Source: J Clin Transl Sci. 2024 Jan 5;8(1):e21. doi: 10.1017/cts.2023.712 (PMC10879999; doi:10.1017/cts.2023.712)
Supplement: Nelson et al. supplementary material 1 — Nelson et al. supplementary material [file S2059866123007124sup001.docx]

# Appendix A: eLearning Course Listing

**Clinical Research Essentials**

Informed Consent Simulation

Good Clinical Practice (GCP) Simulation

Introduction to Clinical Trials

Ethics and Human Subject Protection: A Comprehensive Introduction

Ethics and Human Subject Protection: A Refresher Course

Investigator Responsibilities

The Drug Development Process: Improving Trial Feasibility and Exploring Your Growth Potential

Theory to Practice: Operationalize Your Clinical Study Protocol

Mastering the Event Reporting Cycle: Understanding Your Impact on Patient Safety

Using Metrics to Improve Subject Recruitment and Retention

ICH Gap Analysis

Professional Certification Exam Prep

**Specialized Topics**

Understanding Clinical Trial Protocols: Key Considerations for Effective Development and Feasibility Review

Site Quality Management Tools: SOPs, Metrics, and Training

Risk-Based Monitoring: The Essentials

Mastering Budgeting at Your Site: Building and Negotiating Clinical Trial Budgets that Make Sense

Key Skills for Ensuring Quality Control through Risk-Based Decision-Making

Trial Feasibility and Selection: Their Impact on Accrual

Implementing a Patient-Centered Informed Consent Process

Improving Recruitment, Accrual, and Retention in Clinical Trials

Form FDA 1572: Get it Right the First Time

Inspection Readiness: Best Practices for Managing Clinical Trial Inspections

Building Quality Management Systems for Sites and Sponsors: Root Cause and CAPA

eResearch: Managing Clinical Trials in an Electronic Environment

# Appendix B: Co-Mentoring Interview guide

We would like to learn about your experiences with the co-mentoring circles.

1. In what way has the co-mentoring circle addressed your professional needs? What aspect was the most satisfying? What about certification? Is there more need for the online to take more time or the co-mentoring to take more time? Should there be more emphasis on the online portion?
   1. What did the circles not address? What else were you hoping to achieve through the co-mentoring circles? Anything you wish you could change about the co-mentoring circles, how they're organized with topics abroad, how much time is dedicated to them?
   2. How feasible is it to bring PIs into a training like this? What could be the takeaways that would support you in interacting with PI’s?
2. If you were helping us design the next iteration, the co-mentoring circles 2.0, what could be the curriculum? What might be some ideas or sort of additions to the existing curriculum that would be useful?
3. What aspects of the co-mentoring circles influenced your professional identity? If you think about your professional identity, what aspects of the program make it easy for you to explain, what is it that you do? How that experience translated to where you feel you're in your careers and your professional development identities?
