# Supplementary material for: Pilot implementation of a co-mentoring circles program for the clinical research professionals: Evidence for formative evaluation and logic model
Source: J Clin Transl Sci. 2024 Jan 5;8(1):e21. doi: 10.1017/cts.2023.712 (PMC10879999; doi:10.1017/cts.2023.712)
Supplement: Nelson et al. supplementary material 2 — Nelson et al. supplementary material [file S2059866123007124sup002.docx]

# Appendix B: Co-Mentoring Interview guide

We would like to learn about your experiences with the co-mentoring circles.

1. In what way has the co-mentoring circle addressed your professional needs? What aspect was the most satisfying? What about certification? Is there more need for the online to take more time or the co-mentoring to take more time? Should there be more emphasis on the online portion?
   1. What did the circles not address? What else were you hoping to achieve through the co-mentoring circles? Anything you wish you could change about the co-mentoring circles, how they're organized with topics abroad, how much time is dedicated to them?
   2. How feasible is it to bring PIs into a training like this? What could be the takeaways that would support you in interacting with PI’s?
2. If you were helping us design the next iteration, the co-mentoring circles 2.0, what could be the curriculum? What might be some ideas or sort of additions to the existing curriculum that would be useful?
3. What aspects of the co-mentoring circles influenced your professional identity? If you think about your professional identity, what aspects of the program make it easy for you to explain, what is it that you do? How that experience translated to where you feel you're in your careers and your professional development identities?
